# Increased Frequency of Indels in Hypervariable Regions of SARS-CoV-2 Proteins—A Possible Signature of Adaptive Selection

**DOI:** 10.3389/fgene.2022.875406

**Published:** 2022-06-02

**Authors:** Arghavan Alisoltani, Lukasz Jaroszewski, Mallika Iyer, Arash Iranzadeh, Adam Godzik

**Affiliations:** ^1^ Biosciences Division, School of Medicine, University of California, Riverside, Riverside, CA, United States; ^2^ Graduate School of Biomedical Sciences, Sanford Burnham Prebys Medical Discovery Institute, La Jolla, CA, United States; ^3^ Computational Biology Division, Department of Integrative Biomedical Sciences, University of Cape Town, Cape Town, South Africa

**Keywords:** indels, SARS-CoV-2, protein loop, hypervariable regions (HVR), variants of concern (VOCs)

## Abstract

Most attention in the surveillance of evolving SARS-CoV-2 genome has been centered on nucleotide substitutions in the spike glycoprotein. We show that, as the pandemic extends into its second year, the numbers and ratio of genomes with in-frame insertions and deletions (indels) increases significantly, especially among the variants of concern (VOCs). Monitoring of the SARS-CoV-2 genome evolution shows that co-occurrence (i.e., highly correlated presence) of indels, especially deletions on spike N-terminal domain and non-structural protein 6 (NSP6) is a shared feature in several VOCs such as Alpha, Beta, Delta, and Omicron. Indels distribution is correlated with spike mutations associated with immune escape and growth in the number of genomes with indels coincides with the increasing population resistance due to vaccination and previous infections. Indels occur most frequently in the spike, but also in other proteins, especially those involved in interactions with the host immune system. We also showed that indels concentrate in regions of individual SARS-CoV-2 proteins known as hypervariable regions (HVRs) that are mostly located in specific loop regions. Structural analysis suggests that indels remodel viral proteins’ surfaces at common epitopes and interaction interfaces, affecting the virus’ interactions with host proteins. We hypothesize that the increased frequency of indels, the non-random distribution of them and their independent co-occurrence in several VOCs is another mechanism of response to elevated global population immunity.

## Introduction

Insertions/deletions (indels), are the second most common modifications in the evolution of viral genomes after single nucleotide polymorphisms (SNPs), yet receive relatively little attention in genome analyses (Palmer and Poon, 2019). One of the reasons for that is that their consequences on protein structure and function are more challenging to determine than SNPs. Examples of long, loss-of-function deletions removing entire proteins or functional domains were shown to be deleterious (Zwart et al., 2014) or attenuating (Oostra et al., 2007); however, the effects of shorter, function-refining indels are mostly unknown. Such indels tend to happen in the loops between secondary structure elements, but interestingly not in all the loops, so their distribution cannot be explained by the plasticity of protein structure alone. Such indels rarely affect the overall structure of proteins, but may alter the binding specificity or protein-protein interaction surfaces (Studer et al., 2013), in few studied examples leading to increased drug resistance and immune escape in viruses (Wood et al., 2009; Palmer and Poon, 2019). Their prevalence, evolutionary dynamics, and overall consequences for fitness of most viruses, including SARS-CoV-2, largely remain unacknowledged and unaddressed.

Severe acute respiratory syndrome coronavirus 2 (SARS-CoV-2) first emerged in Wuhan, China and subsequently spread worldwide and infected millions of people in several waves of evolving variants. Its high mutability (van Dorp et al., 2020), typical for RNA viruses (Duffy, 2018) but exacerbated by the scale of the COVID-19 pandemic, has resulted in the emergence of multiple lineages. Higher infectivity, transmissibility and/or lower efficacy of the current vaccines have been reported for Beta (B.1.351*) (Tegally et al., 2021), Gamma (*p*.*) (Jewell, 2021; Madhi et al., 2021), Delta (B.1.617.2, AY. *) (Planas et al., 2021) (Cherian et al., 2021), Lambda (C.37) (Kimura et al., 2022) and Omicron variants (B.1.1.529 and BA.*) (Karim and Karim, 2021; Viana et al., 2021). Tracking and analyzing new emerging lineages with modified disease phenotypes, dubbed variants of concern (VOCs) (Plante et al., 2021), is crucial for determining the strategies of fighting the COVID-19 pandemic. Massive sequencing of SARS-CoV-2, with over 10M genomes available today gives the United States a unique opportunity to study its evolution on the timescale of weeks or even days, as compared to much longer timescales available by comparing species. Much attention has been focused on specific mutations, such as E484K in the spike protein and their effects on host immune response (Starr et al., 2020; Jangra et al., 2021). At the same time, deletions and insertions received less attention, being less frequent, especially in the first phase of the pandemic and more challenging to interpret.

Still, several specific indels in SARS-CoV-2 in the envelope protein (Kumar et al., 2021), non-structural protein 1 (NSP1) (Lin et al., 2021), spike glycoprotein (spike or S) (McCarthy et al., 2021) and accessory ORFs (Lam et al., 2020), have been studied in detail. The NSP1 Δ79-89 was shown to be associated with lower IFN-β levels and non-severe phenotypes (Lin et al., 2021). Our analysis presented here expands on these examples and provides an overview of the dynamics of in-frame indels in the evolution of the SARS-CoV-2 genome. Regions with recurrent indels called recurrent deletion regions (RDRs) and recurrent insertion regions (RIRs) in the N-terminal domain (NTD) of the spike were shown to play a role in immune escape (McCarthy et al., 2021). Here we use the term hypervariable regions (HVRs) to refer to indel-prone regions. These concentrations of indels provide an example of a new paradigm of the effects of indels on viral genomes and proteins—instead of loss-of-function they modify it by remodeling protein surfaces, affecting major antibody epitopes (Cai et al., 2021) and, possibly, protein-protein interaction networks.

## Methods

### SARS-CoV-2 Sequencing Data Collection

We retrieved multiple sequence alignment (MSA) and metadata of complete SARS-CoV-2 genomes (6,143,793) from GISAID (https://www.gisaid.org/) as of January 7^th^, 2022. Briefly, full alignment (msa_0106.fasta) provided by GISAID was based on 6,716,124 submissions to GISAID EpiCoV. GISAID pipeline excludes duplicate, low-quality sequences (>5% N content) and incomplete sequences (length <29,000 bp). Then, the GISAID pipeline used this cleaned data to create the MSA file of 6,143,793 sequences using MAFFT (Katoh and Standley, 2013) with hCoV-19/Wuhan/WIV04/2019 (EPI_ISL_402,124; GenBank: MN996527) used as reference (Zhou et al., 2020).

### Identification of Indels

We used an in-house Perl script to identify variations in each genome based on the GISAID MSA file as of January 7^th^, 2022. Additionally, on top of GISAID’s cutoffs for excluding low-quality genomes with high N content (0.05), we applied additional filtering to avoid spurious indels and indels with shifted positions arising from high N content. Moreover, genomes with more than 200 mutations were excluded, resulting in 4,976,200 SARS-CoV-2 genomes used in the downstream analysis in this study. Additionally, to avoid reporting spurious indels arising from sequencing errors or errors in MSA, we generated another MSA file with no gaps in reference (obtained with *keep reference length* option) (Katoh and Standley, 2013) to confirm the exact positions of all the deletions discussed in this study. Then, for visualizing and confirming the position of the indels we used the MSA file based on a representative genome for each of the indels with 0 N content.

### Assessing Differences in the Rate of Indels Between SARS-CoV-2 Proteins

We adopted the method we recently used to identify significantly under-mutated and over-mutated proteins during SARS-CoV-2 evolution (Jaroszewski et al., 2021) to identify proteins with a high rate of indels. Briefly, we counted the total number of indels (except single residue deletions which are usually regarded as unreliable) for each protein (except NSP11, ORF3b, ORF9b and ORF14 as these are too short for the significance analysis). We then used a two-sided binomial test to compare the rate of indels in each protein to the rate of indels in the background (all proteins) to identify proteins with high rates of indels. Our previous study (Jaroszewski et al., 2021) showed that ORF1ab is less frequently mutated and is likely under more stringent purifying selection than the genes coding for structural and accessory proteins (ORFs2-10). Therefore, we applied an additional statistical comparison of indel rates to non-structural proteins to identify NSPs (NSP1- NSP16) with a higher rate of indels than others. We performed a separate two-sided binomial test using only ORF1ab (corresponding to proteins NSP1-NSP16) for this specific comparison as background. Adjusted *p*-values (q-values) were calculated using the false discovery rate (FDR) method. Proteins with odds ratio above one and q-values less than 0.01 were considered as having significantly increased rates of indels.

### Visualization of Indels on Proteins’ 3-Dimensional (3D) Structures

We used PyMol (PyMOL, 2021) and Coronavirus3D (Sedova et al., 2020) for studying and visualization of indels in the context of protein 3-dimensional (3D) structures. The 3D coordinates were downloaded from the Protein Data Bank (PDB) (Berman et al., 2000). For proteins with no available 3D structures we used, if available, models predicted by Alphafold (https://deepmind.com/research/open-source/computational-predictions-of-protein-structures-associated-with-COVID-19), or homology modeling (https://zhanglab.dcmb.med.umich.edu/COVID-19/), noting in the discussion their hypothetical status. It should be noted that even for some proteins with available 3D structures we used models predicted with homology modeling when the indels were located in the regions of the protein with unresolved structures (unmodeled residues). Information on protein domain boundaries was based on 3D coordinates when available or on UniProt and the literature (Supplementary Table S4).

The positions of transmembrane helices for proteins with no available 3D structures were identified with the TMHMM 2.0 algorithm (Krogh et al., 2001). IEDB server (Bepipred Linear Epitope Prediction 2.0 at http://www.iedb.org/) (Jespersen et al., 2017) was used to predict B-cell epitopes for NSP1, NSP3, NSP6, spike, nucleocapsid, ORF3a, ORF7a, and ORF8 (i.e. proteins with significantly increased rates of indels).

### Visualization of Indels on the Phylogenetic Tree

We mapped the number of indels for each genome (between one and six indels) on the Nextstrain time-resolved tree (Hadfield et al., 2018), which includes 3475 genomes sampled between December 2019 and Dec 27^th^, 2021. We used the ggtree R package (Yu, 2020) to visualize the tree.

### Visualization of Indels on the Alignment File

We extracted one representative genome for each of the indels discussed in this study (i.e., the indels most frequently observed in SARS-CoV-2 genomes). These genomes were then used to visualize the indels using R packages ggmsa and Biostrings.

### Analysis of Independent Occurrence of Indels in SARS-CoV-2

The independent acquisition of indels was determined using HomoplasyFinder (Crispell et al., 2019) with the same filtering criteria as used in the previous studies (van Dorp et al., 2020). To identify potential recurrent indels (independently acquired in different branches of phylogenetic tree) in SARS-CoV-2 genomes, we used the GISAID global tree that includes 4,701,022 SARS-CoV-2 genomes (GISAID as of January 7^th^, 2022) (Shu and McCauley, 2017) together with the input variant calling file (VCF). Briefly, HomoplasyFinder calculates the consistency index for each indel by dividing the minimum number of changes on the GISAID tree (MNCT) by the number of different indels observed at that site minus one. The most frequent indels (observed in at least 0.01% of all studied genomes) with a consistency index of <1 and MNCT >30 were reported as potentially recurrent indels if they were also independently acquired in more than two independent GISAID clades and in at least two PANGO lineages when their immediate ancestor didn’t carry this indel, two-time points and two different continents (Originating lab). These filtering and stringent cutoffs were applied to address issues arising from mixed quality of assembled genomes, which in some cases are not detectable (e.g., assembly pipelines replace missing nucleotides with data from the reference genome) from the genome analysis alone. The quality issues introduce uncertainty in phylogenies, lineage assignments and underestimation of indels frequencies all lead to overestimation of independent occurrence of indels (De Maio et al., 2020; Turakhia et al., 2020; Tang et al., 2021), which we countered by increasing the cutoff thresholds. Regions with different recurrent indels which occurred in adjacent residues (up to five residues apart) were called hypervariable regions (HVRs). The HVRs observed in this study contain between 2 and 30 residues.

To calculate the recurrence of each indel as the function of time of sample collection, geographical location (originating lab), PANGO lineages, and GISAID clades, we grouped genomes into 25-time bins based on the month and year of the data collection, into six geographical locations (continents), 12 clade-based groups (G, GH, GK, GR, GRA, GRY, GV, L, O, S, V, and a non-assigned group), and 1544 different PANGO lineages. We used such relatively large groups to reduce noise arising from the difference between individual labs and from low-quality genomes.

### Statistical Analysis of Co-Occurred Indels in SARS-CoV-2 Genomes

We ran cooccur R package to analyze the co-occurrence of indels in each lineage and all genomes and used ggplot2 R package (Wickham, 2011) to draw heatmap of correlation matrix. We also calculated Spearman’s correlation coefficient and *p*-value of the correlation test for every two indels using hmisc (Harrell and Harrell, 2019) R package. We further checked the independent acquisition of top correlated/co-occurred indels using HomoplasyFinder (Crispell et al., 2019) based on the method explained earlier. The input VCF file includes information on the presence/absence of two co-occurred indels. We used ComplexHeatmap R package (Gu et al., 2016) to draw the heatmap of percentage of top indels in SARS-CoV-2 VOCs.

### Comparing SARS-CoV-2 and SARS-CoV Genomes in Terms of Indels

Spike, NSP1, NSP3, NSP6, N, ORFs 3a, 7a, and eight protein sequences of SARS coronavirus Tor2 (NC_004,718.3) and SARS-CoV-2 (MN996527) were aligned using MAFFT (Katoh and Standley, 2013) (default parameters). We used Jalview (Waterhouse et al., 2009) to visualize alignment files and obtain the count and positions of indels.

## Results

### Increased Frequency of In-Frame Indels in Emerging SARS-CoV-2 Lineages

**FIGURE 1:**
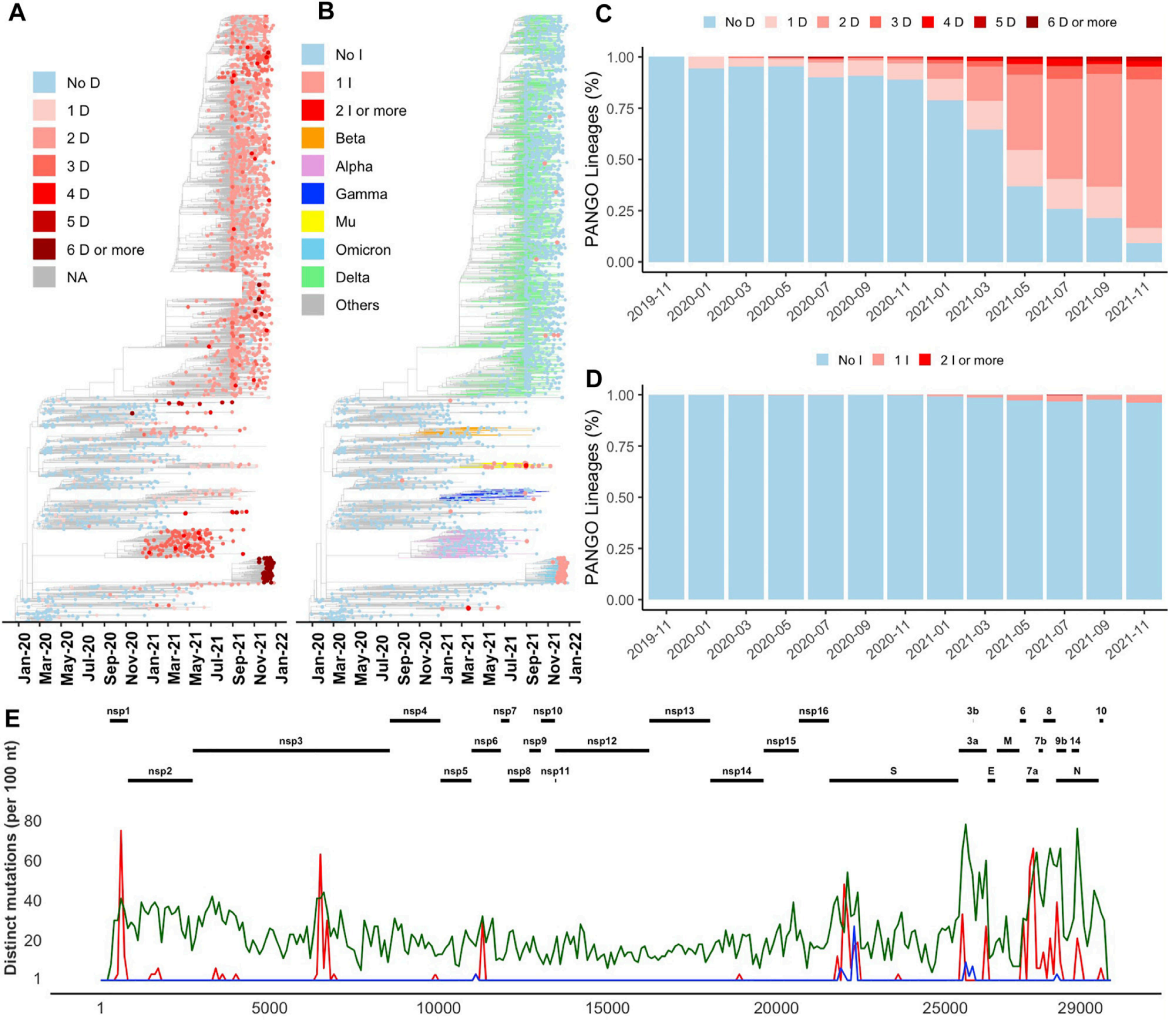
Distribution of indels in SARS-CoV-2 genomes **(A)** and **(B)** Increase in the number of deletion **(D)** and insertion **(I)** events in newly emerged lineages illustrated on Nextstrain’s time-resolved phylogenetic tree, respectively **(C)** and **(D)** Percentage of PANGO lineages with and without deletion and insertion events over time, respectively **(E)** Distribution of the most common deletions along the SARS-CoV-2 genome (red) compared to insertions (blue) and missense substitutions (green).

The recent increase in the number of indels (both insertions and deletions) was observed in all branches of the phylogenetic tree (Figures 1A,B **)**. This increase can be seen in the percentage of both SARS-CoV-2 lineages (Figures 1C,D **)** and genomes (Supplementary Figures S1A,B) with at least one deletion or one insertion event (one or more than one amino acid change) growing in time. Indels were acquired in several VOCs such as Alpha (B.1.1.7 and Q.*), Beta (B.1.351*) and Omicron (B.1.1.529 and BA.*), Gamma (P.*), and Delta (B.1.617.2, AY.*). As an example, Alpha variant (B.1.1.7) is defined by 17 signature genome modifications, including three deletions events (NSP6 Δ106–108, S Δ69-70, and S Δ144), while Omicron variant includes seven indels as shown in Supplementary Figure S1C (NSP3 1265:SL>I, NSP6 Δ105-107, nucleocapsid Δ31–33, S Δ69–70, S 142:GVYY>D, S 211:NL>I, and S 214:R>REPE). Additional indels and their combinations are found in other variants (Supplementary Figure S1C). In this study, for simplicity, genome modifications that include both indels and substitutions such as S 142:GVYY>D are only referred to as indels.

### Indels are concentrated on protein surfaces near epitope regions

Most indels are significantly (q-value < 0.01 and odds ratio >1) concentrated in NSP1, NSP3, NSP6, ORF3a, ORF6, ORF7a, ORF7b, ORF8, nucleocapsid, and spike glycoprotein (Figure 1E and Table 1), all of which are involved in interactions with the host immune system (Lei et al., 2020; Liang et al., 2021; Smith et al., 2021). At the same time, proteins involved in the replication–transcription complex show very few or no indels (Figure 1E and Table 1). It is in agreement with our earlier report showing the segment of the genome coding for the non-structural proteins (Orf1ab, corresponding to proteins nsp1-nsp16) is significantly under-mutated for both missense and synonymous mutations (Jaroszewski et al., 2021). It should be noted that terms recurrent deletion regions (RDRs) and recurrent insertion regions (RIRs) are used in recent literature, indicating regions of SARS-CoV-2 proteins with frequent recurrent deletions and insertions, respectively. In this paper, we use the term “hypervariable regions (HVR)” referring to regions of proteins with frequent recurrent indels.

**TABLE 1:** Comparison of frequencies of in-frame indels (indels) in SARS-CoV-2 proteins using the two-sided binomial test (only indels observed in at least two genomes were included to eliminate spurious mutations). Bold font indicates proteins with a significantly increased rate of indels (q-value<0.01 and Odds ratio>1).

Protein	Protein length	Number of indels	All Proteins as Background		ORF1ab as Background	
			Odds Ratio	q-value (FDR adjusted *p*-value)	Odds Ratio	q-value (FDR adjusted *p*-value)
NSP1	540	109	2.14	1.85E-12	4.40	1.44E-36
NSP2	1914	81	0.45	5.89E-17	0.92	5.02E-01
**NSP3**	**5835**	**442**	**0.80**	**1.78E-07**	**1.65**	**3.96E-32**
NSP4	1500	40	0.28	5.58E-24	0.58	2.93E-04
NSP5	918	6	0.07	3.74E-29	0.14	9.58E-12
**NSP6**	**870**	**58**	**0.71**	**6.47E-03**	**1.45**	**8.99E-03**
NSP7	249	5	0.21	1.18E-05	0.44	6.23E-02
NSP8	594	9	0.16	1.86E-14	0.33	1.65E-04
NSP9	339	7	0.22	2.31E-07	0.45	3.54E-02
NSP10	417	8	0.20	4.37E-09	0.42	1.08E-02
NSP12	2795	46	0.17	2.91E-64	0.36	5.63E-18
NSP13	1803	15	0.09	3.32E-54	0.18	2.65E-20
NSP14	1581	91	0.61	2.58E-07	1.26	3.54E-02
NSP15	1038	36	0.37	1.05E-12	0.76	9.92E-02
NSP16	894	23	0.27	6.51E-15	0.56	5.01E-03
**Spike**	**3822**	**459**	**1.27**	**1.22E-07**	**-**	**-**
E	228	18	0.84	5.16E-01	-	-
M	669	26	0.41	2.06E-07	-	-
**N**	**1260**	**159**	**1.34**	**3.43E-04**	-	-
**ORF10**	**117**	**7**	**0.63**	**3.01E-01**	-	-
**ORF3a**	**828**	**254**	**3.25**	**1.91E-57**	-	-
**ORF6**	**186**	**61**	**3.47**	**9.43E-16**	-	-
**ORF7a**	**366**	**595**	**17.22**	**0.00E+00**	-	-
**ORF7b**	**132**	**58**	**4.65**	**1.56E-20**	-	-

**FIGURE 2:**
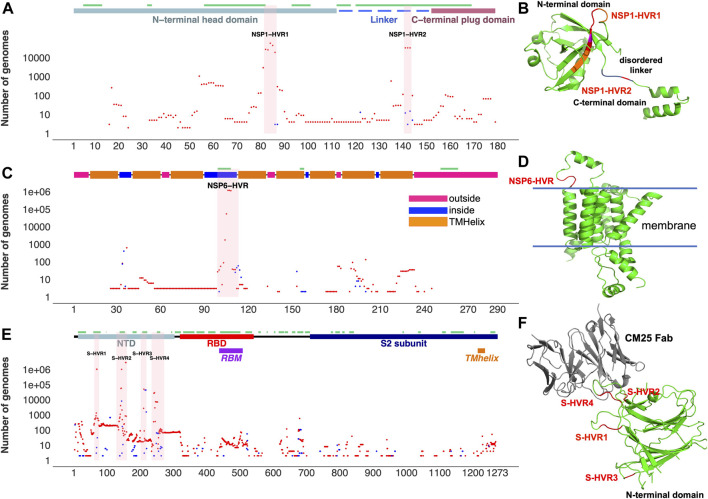
Top SARS-CoV-2 HVRs in the context of protein 3D structures. **(A)** Distribution of indels in NSP1 **(B)** NSP1-HVRs on protein 3D structure **(C)** Distribution of indels in NSP6 **(D)** NSP6-HVR on protein 3D structure **(E)** Distribution of indels in spike glycoprotein **(F)** HVRs on the protein 3D structure of the spike glycoprotein N-terminal domain bound to human Fab CM25. Insertions, deletions, and predicted B-cell epitopes (result from the IEDB server at www.iedb.org) are represented as blue dots, red dots, and green lines, respectively. Supplementary Table S3 provides details of structures/models used in the Figure.

**FIGURE 3:**
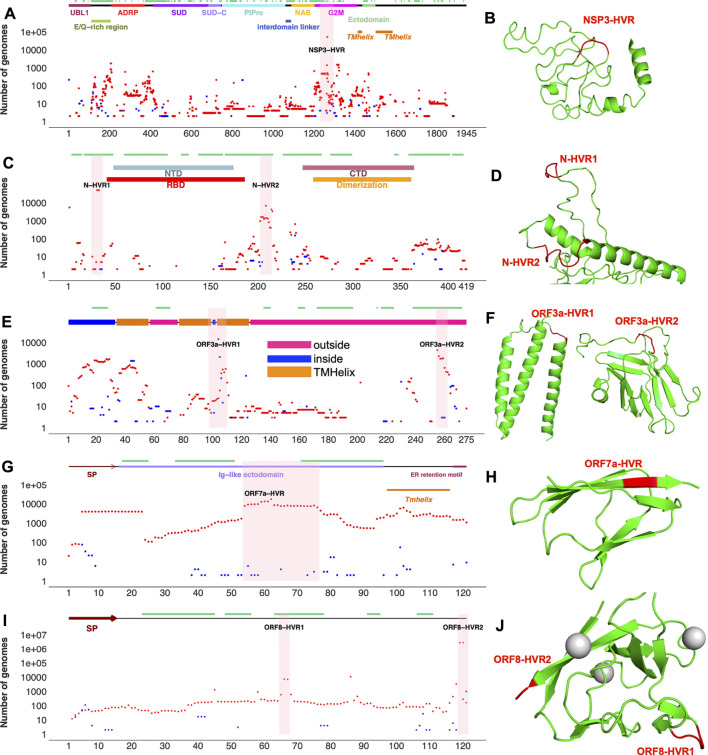
Top SARS-CoV-2 HVRs in the context of protein 3D structures **(A)** Distribution of indels in SARS-CoV-2 non-structural protein 3 (NSP3) **(B)** NSP3 recurrent deletion region (HVR) on protein 3D structure **(C)** Distribution of indels in SARS-CoV-2 nucleocapsid (N) protein **(D)** N-HVRs on protein 3D structure **(E)** Distribution of indels in SARS-CoV-2 ORF3a **(F)** ORF3a-HVRs on protein 3D structure **(G)** Distribution of indels in SARS-CoV-2 ORF7a **(H)** ORF7-HVR on protein 3D structure **(I)** Distribution of indels in SARS-CoV-2 ORF8 **(J)** ORF8-HVRs on protein 3D structure. Deletions, insertions, and epitopes are represented as red dots, blue dots, and green lines, respectively. Pink highlighted regions represent HVRs or potential hotspots for recurrent indels in each protein. The regions of 3D structure corresponding to HVRs are colored in red. The coordinates of proteins were obtained from different sources (see Supplementary Table S3). Predicted 3D structural models https://zhanglab.ccmb.med.umich.edu/COVID-19/ were used for visualization of recurrent deletion regions in NSP3, ORF3a, and nucleocapsid protein. SP: signal peptide. Indels independently occur in several SARS-CoV-2 lineages in hypervariable regions.

Aggregation and recurrence of indels in hypervariable regions of SARS-CoV-2 proteins are determined by an interplay of the protein structural constraints and functional role of specific regions. Most of the HVRs of SARS-CoV-2 proteins (except ORF7a-HVR) are found on or adjacent to loops forming either experimentally identified (Liang et al., 2021; Smith et al., 2021) or predicted antibody epitopes (Figures 2,3), suggesting SARS-CoV-2 is optimizing its interactions with the host immune system, possibly in response to the increased immunity of the population. For instance, NSP6-HVR falls on a predicted T-cells (Smith et al., 2021) and B-cells epitope (per IEDB server), forming a short loop between two transmembrane helices (Figure 2C). Similarly, NSP1-HVR1 and spike-HVRs (Figure 2), as well as HVRs in other proteins are in or near the loop forming epitope regions (Figure 3).

In the most studied SARS-CoV-2 protein, surface glycoprotein S (spike), NTD is one of the most genetically modified regions of spike protein and of the entire SARS-CoV-2 proteome (see Figure 1). Deletions in the NTD could classified as belonging to recurrent deletion regions: RDR1 (residues 60–75), RDR2 (residues 139–146), RDR3 (residues 210–213), and RDR4 (residues 242–248) (McCarthy et al., 2021). Recurrent insertions were also reported in the same regions (Gerdol, 2021). We observed that indels in NTD-HVR1 and HVR2 are more frequent as compared to HVR3 and HVR4 (Supplementary Figure S2A,B). Several lineages with new spike indels (expanding spike-HVR2 and HVR4) are now emerging (Supplementary Figure S2A,B). Comparison of spike proteins from the SARS-CoV (Tor2) and SARS-CoV-2 (one of the early Wuhan reference) viruses indicates 22 amino acid (AA) insertions and four AA deletions in SARS-CoV-2 spike protein compared to SARS-CoV that mainly occurred in NTD (Supplementary Figure S2C), confirming that NTD is generally the most indel-prone region of spike in SARS coronaviruses.

**FIGURE 4:**
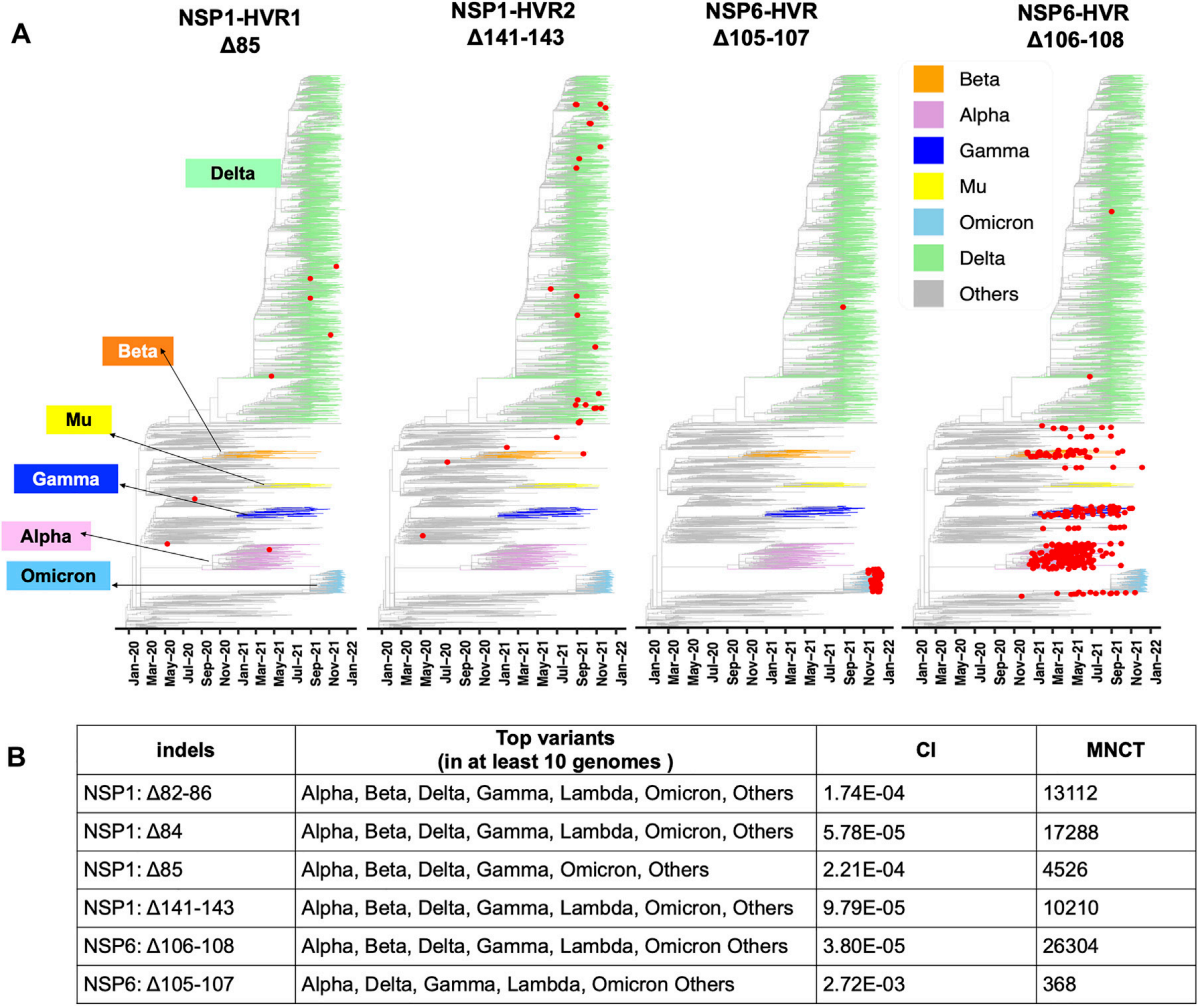
Recurrent Indels in NSP1 and NSP6. **(A)** Nextstrain time-resolved tree, which includes 3475 genomes sampled between December 2019 and Dec 27^th^, 2021) displays the presence and distribution of the most frequent deletions positioned on NSP1-HVRs and NSP6-HVR as red dots **(B)** Top SARS-CoV-2 variants harbor the most frequent and potentially recurrent deletions of NSP1 and NSP6. Minimum Number of Changes on Tree (MNCT) and Consistency Index (CI) calculated using HomoplasyFinder based on GISAID global tree (4,701,022 SARS-CoV-2 genomes as of January 7^th^, 2022).

NSP3 HVR corresponds to group 2 specific marker domain (G2M), a structurally uncharacterized region of the protein (Figures 3A,B). Based on the NSP3 predicted model (built using D-I-TASSER/ C-I-TASSER pipeline from the Zhang lab, https://zhanggroup.org/), NSP3-HVR is in the loop and indels in this region occur near B-cell epitopes predicted using IEDB server (Figure 3B). Similar observations were also made for nucleocapsid protein (Figure 3 C,D), ORF3a (Figure 3 E,F), and ORF8’s HVR (Figures 3I,J). The indels in different protein HVRs occurred independently in several lineages (Figure 4 and Supplementary Table S1) as seen on the SARS-CoV-2 phylogenetic tree (Elbe and Buckland-Merrett, 2017). In the following, we will discuss in detail the independent acquisition of indels in NSP1, NSP6 and NTD of spike protein HVRs. Independently acquired indels in NSP3, ORF3a, ORF7a, and ORF8 as well as in nucleocapsid protein HVRs will be discussed in separate sections.

The independent acquisition of indels was determined using HomoplasyFinder (Crispell et al., 2019) with filtering criteria as applied in the previous study (van Dorp et al., 2020). Indels with minimum number of changes on tree (MNCT) above 30 were considered as potential recurrent deletions. We then applied additional filters (see above) and only included those that fulfilled all the criteria (Supplementary Table S1). These stringent cutoffs were applied to avoid overestimation of homoplasies due to sequencing errors (De Maio et al., 2020).

**FIGURE 5:**
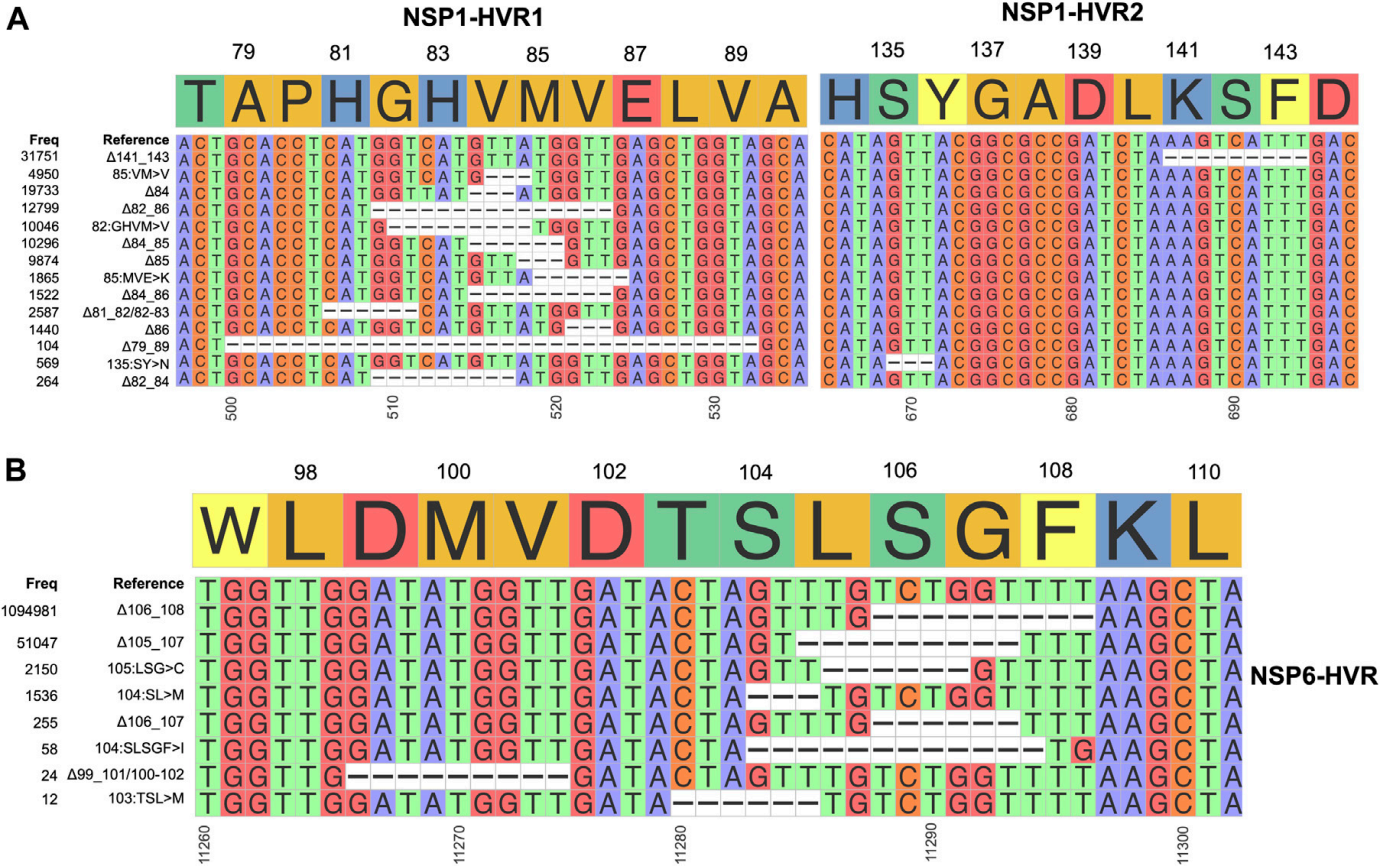
Hypervariable regions (HVRs) of NSP1 and NSP6 **(A)** and **(B)** represent coordinates of HVRs of NSP1 and NSP6, respectively. The number of genomes containing a specific indel is provided on the left side of each plot. Indels independently co-occur in several SARS-CoV-2 lineages.

Two mutually exclusive NSP1 HVRs (e.g., NSP1 Δ84 and NSP1 Δ85 in NSP1-HVR1 and Δ141-143 in NSP1-HVR2) emerged independently in several lineages such as Alpha, Beta, Delta, Gamma and Omicron (Figures 4 A, B). A long version of the indel in NSP1-HVR1 (Δ79-89) was studied before (Lin et al., 2021), but our analysis indicates that shorter indels in this region are recurring more frequently (Figure 5A **)**. The results from HomoplasyFinder (consistency index or CI) indicate that NSP1 deletions are among the potential recurrent events in SARS-CoV-2 evolution (Figure 4B and Supplementary Table S1). NSP1 (Δ79-89) was reported to induce lower IFN-I response in the infected Calu-3 cells (Lin et al., 2021), highlighting the biological importance of indels in NSP1 and other non-spike proteins. It should be noted that NSP1 deletions are not among signature genomic modifications of any SARS-CoV-2 lineage and no indel event differences were identified between NSP1 proteins of SARS-CoV (Tor2) and SARS-CoV-2 (Supplementary Figure S3A). This might imply that intact NSP1 is key for the full functionality of the virus and its pathogenicity but at the same time recurrent indels could suggest the presence of intra-host variations and quasispecies (Santacroce et al., 2021).

After the spike-HVRs, the NSP6-HVR (residues 99–108) is the second most frequently modified HVR in SARS-CoV-2, with the Δ106-108 observed in more than 1M genomes as of January 2022 (Figure 5A). NSP6 deletions independently occurred as a signature modification for several VOCs—Alpha, Beta, Gamma, and Omicron but also some other lineages such as B.1.525 in Nigeria and Europe and B.1.526 in New York and Europe (Figure 4 and Supplementary Table S2). Signatures of positive selection for NSP6 Δ106-108 were recently reported (Martin et al., 2021) in line with our results showing high recurrence of NSP6 deletions (Figure 4B). In addition to recurrent indels, overlapping indel events identified in NSP1 (Figure 5A), NSP6 (Figure 5B), and Spike NTD (Supplementary Figure S2) could provide additional evidence of convergent and/or parallel adaptive evolution in SARS-CoV-2 genomes. This may also offer more potential genetic routes for the rapid adaptation, immune escape and drug resistance of SARS-CoV-2. Similar evolutionary routes in HIV-1 and other RNA viruses were found to play pivotal role in drug and neutralizing antibody resistance (Menéndez-Arias et al., 2006; Gutierrez et al., 2019).

**FIGURE 6:**
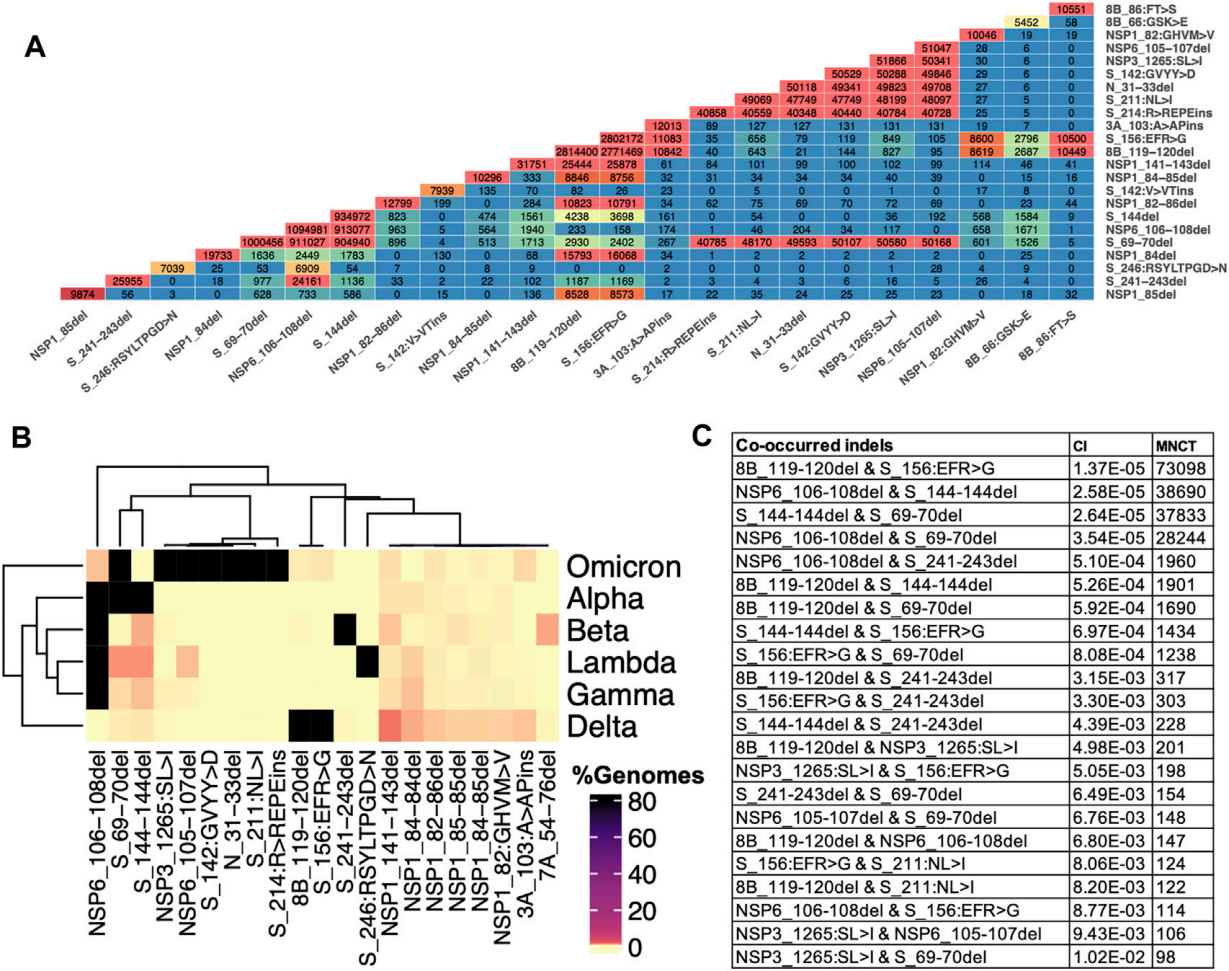
Indels and their co-occurrence in SARS-CoV-2. **(A)** Co-occurrence of top frequent indels **(B)** Co-occurrence of top indels in VOCs. Data for these heatmaps is provided in Supplementary Table 2 which includes additional combinations of indels in lineages harboring them **(C)** Independent co-occurrence of indels determined based on minimum number of changes on tree (MNCT) and consistency index (CI) calculated using HomoplasyFinder based on GISAID global tree (4,701,022 SARS-CoV-2 genomes as of January 7th, 2022).

We observe an increasing number of genomes with two or more different indels in spike or other proteins. We use the term co-occurred indels for indels that appear simultaneously in at least one SARS-CoV-2 genome, and independent acquisition of top co-occurred indels was determined using HomoplasyFinder (see method section for details). Multiple spike-indels independently co-occurred with each other and with indels in other proteins, especially NSP6-indels (Figure 6 and Supplementary Table S2). NSP6-indels independently co-occurred with spike indels located in HVR1 and HVR2 in Alpha (B.1.1.7, Q.*) and B.1.525, with indels located in HVR2 in B.1.526.1 and B.1.1.318, with indels in HVR4 in Beta (B.1.351*) and with several indels in HVR2 and HVR3 in Omicron (B.1.1.529 and BA.*) as shown in Figure 5 and Supplementary Table S2. Based on HomoplasyFinder results, indels in the spike NTD and ORF8 are also among the top co-occurred indels. Spike Δ157-158 and ORF8 Δ119-120 were found in more than 90% of the genomes assigned to Delta variant and their co-occurrences were also recorded in genomes assigned to other lineages such as Omicron and B.1.485 (Figure 6B and Supplementary Table S2).

### Hypervariable Region in SARS-CoV-2 Non-Structural Protein Three NSP3

NSP3 along with NSP1 and NSP6 has significantly higher number of indels when compared to the rest of NSPs (Table 1). As shown in Figure 3, indels in NSP3 are largely occurring in the loop region (1235–1270) and near epitopes (Smith et al., 2021). NSP3 deletion 1265:SL>I is a signature mutation of Omicron variant, NSP3 Δ1237-1251 was observed in L.1 PANGO lineage in Canada and NSP3 Δ1263 in B.1.1.298 variant from Denmark where the latter co-occur with NSP1 85:VM>V and spike Δ69-70. NSP3-indels are often mutually exclusive with indels in other proteins - they only co-occurred with spike and NSP6-indels in Omicron and very few genomes assigned to B.1.1.7 lineage (Supplementary Table S3). When compared to NSP3 of SARS-CoV, SARS-CoV-2 NSP3 had a total of 30 AA insertions and seven AA deletions which occurred mostly between residues 100–400 (Supplementary Figure S4) correspond to predicted epitopes (Supplementary Figure S2 **).**

Although NSP2 was not identified as a significantly indel-prone protein, some indels in the NSP2 appeared independently in several lineages (Supplementary Tables S1, S2 **)**. The NSP2 Δ265-266 is the signature modification of the B.1.573, B.1.1.191, and AN.1 PANGO lineages (Supplementary Table S2), primarily seen in Canada and Denmark samples. The NSP2 Δ268 is mainly occurring in viral genomes collected from England, Scotland, Northern Ireland, and the Netherlands, and it is also the signature mutation of several lineages (Supplementary Table S2). The NSP2 Δ267-268 frequently appeared during the early phase of the pandemic and only a small portion of the recently collected genomes harbored other NSP2-indels positioned on NSP2-HVR (residues 260–270). NSP2 was shown to disrupt host signaling, and it might play a role in SARS-CoV-2 pathogenicity. However, more investigation is required to elucidate the role of NSP2 protein and the impact of its indels on immune evasion.

### Recurrent Deletion Regions in SARS-CoV-2 Nucleocapsid and Accessory Proteins ORF3a, ORF7a, and ORF8

Indels of the nucleocapsid protein occur in two potential HVRs (HVR1: clusters around residues 28–35 and HVR2: clusters around residues 202–214) as shown in Figures 3C,D. Both nucleocapsid HVRs specially HVR-2 are close to experimentally identified epitopes such as 36-RSKQR-40 and 206-SPARM-210 (Liang et al., 2021; Smith et al., 2021). After Omicron signature deletion (Δ31-33 at HVR1) the second most frequent deletion in nucleocapsid protein, 208AR>G (HVR2), is a signature of B.1.1.318 and is found in some B.1.1.7 genomes (Supplementary Table S2). It co-occurred with three other indels in B.1.1.318, including NSP6 Δ106-108, spike Δ144, and ORF7b 44:TNMKF>Y. According to the Coronavirus3D (Sedova et al., 2020) variant tracker, this lineage was among the top growing lineages in several countries such as the United States, United Kingdom, and France in June 2021.

The most recurrent indels of ORF3a cluster around amino acid positions 103 (ORF3a-HVR1) and 255 (ORF3a-HVR2) as shown in Figures 3E,F. ORF3a-HVRs are located in the structurally unresolved region of the protein. Based on the predicted structures, they correspond to loops which also contain predicted B-cell epitopes. Interestingly ORF3a-HVRs identified in our study are also near experimentally identified epitopes of ORF3a antibodies such as 100-GLEAPFLYLYALVYF-114 (Smith et al., 2021), 266-EPTTTTSVPL-275, 246-IHTID-250, and 266-EPTTTTSVPL-275 (Liang et al., 2021).

The only insertion (240P>PE) in ORF3a SARS-CoV-2, when compared to SARS-CoV is located near ORF3a-HVR2 (Supplementary Figure 3D and Figures 3E,F). Despite recurring in several lineages, ORF3a indels are not signature mutations for any lineages or sub-lineages. ORF3a Δ255 co-occurred with NSP6 and spike indels in Alpha variant (Supplementary Table S2 **)**.

Unlike the rest of SARS-CoV-2 proteins, accessory proteins (ORF7a and ORF8) have longer indels. The indels of ORF7a often happen in ORF7a-HVR encompassing residues 60–100 (Figure 3 G,H), near previously identified ORF7a epitopes such as 86-LFIRQEEVQELYSPI-100 (Liang et al., 2021). The most frequent indel in this region is 7A_62:QF>H co-occurred with NSP6 and spike indels in the Delta variant (Supplementary Table S2). ORF7a indels are not signature mutations of any SARS-CoV-2 lineage and protein is mostly conserved between SARS-CoV and SARS-CoV-2 when compared to ORF8 (8b) as shown in Supplementary Figures S3E,F.

The most recurrent and frequent indels of ORF8 is encompassing residues 63–66 (ORF8-HVR1) and 118–120 (ORF8-HVR2) as illustrated in Figure 3 I,J. and the latter is the signature mutation for the Delta variant and co-occurred with spike S_156:EFR>G (Figure 6). Interestingly, both ORF8 HVRs are near experimentally identified epitopes, including 66-GSKSP-70 and 106-EDFLE-110. The highest number of changes in terms of indels between SARS-CoV and SARS-CoV-2 proteins was recorded for ORF8 (8b) and spike proteins (Supplementary Figure S3), indicating they are rapidly evolving among SARS coronaviruses. Deletions of an entire ORF8 were identified during both early and late phases of SARS-CoV pandemic (2003) in China (Consortium, 2004).

Interestingly, most SARS-CoV-2 proteins have a high tendency for recurrent deletions (Supplementary Table S1), likely facilitating the virus adaptation to the human host. The increasing number of deletions also results in SARS-CoV-2 genome shrinkage over time, especially in the recent VOCs like Omicron (Supplementary Figure S4). Although the direct association of genome size with viral fitness is difficult to prove, there is evidence of replicative advantage associated with smaller genome size in RNA viruses (Tromas et al., 2014; Zwart et al., 2014; Walker et al., 2015). The results of this study should be interpreted within the context of limitations in the quality of SARS-CoV-2 genomes. Mixed quality of genomes and high numbers of Ns increases instability in lineage assignments and might underestimate indels and overestimate homoplasies. We accounted for this problem by using very stringent criteria and we hypothesize that the real extent of homoplasy in the SARS-CoV-2 evolution is likely to be even higher.

## Discussion

Viruses, and in particular RNA viruses, are known to undergo rapid genome modifications, but are rarely studied with frequency that would allow us to monitor their detailed dynamics. Comparison of genomes of separate species gives us only a summary of modifications that occurred over significant periods of time. The COVID-19 pandemic led to an unpreceded mobilization of the research community, which in turn provided a unique opportunity for real-time monitoring of a pathogenic virus during a pandemic. In this study, we used sequencing data provided by thousands of research groups and available in a GISAID database (Shu and McCauley, 2017) to study the dynamics of protein indels during the course of pandemic. This analysis revealed the increase in the rate of indels that started in late 2020, driven by the emergence of lineages containing deletions as signature genome modifications, such as Alpha and Beta variants which replaced most of the previous lineages without indels. These were in turn replaced by the Delta variant with even more deletions in its genome. The Omicron variant that appeared in November 2021 is the first VOC containing both insertions and deletions and it has currently replaced almost all previous variants. Some of the indels in these variants were already shown to increase immune invasion, lead to higher transmissibility and higher viral binding affinity (Karim and Karim, 2021; McCarthy et al., 2021; Viana et al., 2021), functions of others are still unknown, but we can speculate about them based on the co-occurrence and overlap with mutations at the same sites.

Different processes may contribute to the emergence of indels in viral genomes, such as replication slippage, recombination, and retrotransposition. Compared to recombination and retrotransposition, replication slippage generates short indels (Viguera et al., 2001; Domingo, 2020). Since our analysis revealed mainly short indels, we believe these indels are primarily the result of replication slippage. Another possible explanation for this hypothesis is that insertions emerged later in the pandemic consistent with a higher evolutionary cost for insertions than deletions due to higher probability of incidence of the slippage-induced deletions.

Regardless of the cause of their emergence, SARS-CoV-2 indels that were selected by evolution and contributed to the emerging lineages are predominantly found in specific regions of proteins known as hypervariable regions that typically correspond to loops in protein structures. Interestingly, not all loops in SARS-CoV-2 proteins were found to contain indels, those that do were close to either experimentally identified or predicted epitopes (Zhang et al., 2008; Liang et al., 2021; Smith et al., 2021) or were involved in protein-protein interactions, and in the case of the specific SARS-CoV-2 proteins with overabundant indels, in interactions with the host’s immune system. Modeling and emerging experimental evidence (Cai et al., 2021) shows that deletions in such regions can remodel epitope surfaces, leading to immune escape. This parallels findings in HIV-1 where deletions in the spike glycoprotein regions encoding surface-exposed disordered loops were found to mediate escape from the neutralizing antibodies elicited by earlier variants of the virus (Wood et al., 2009; Palmer and Poon, 2019).

Many indel-prone regions such as the loops in the spike NTD overlap with mutation hotspots that are thought to be driven by host immune system pressure (Gerdol, 2021; McCallum et al., 2021; McCarthy et al., 2021). Therefore, we hypothesize that the emergence of indels in the same hotspots is a response to the same adaptive pressure. This is supported by the recent studies where both spike-NTD substitutions and indels were demonstrated to accelerate virus adaptation to the host and immune escape (Gerdol, 2021; McCallum et al., 2021; McCarthy et al., 2021).

Independent co-occurrence of indels in several VOCs might reflect signatures of adaptive evolution by recurrence or recombination. Several VOCs such as Alpha, Beta and Omicron which have simultaneous spike and NSP6-indels were found to have higher transmissibility, infectivity, or immune escape properties than the previously dominant lineages such as B.1.177 (Davies et al., 2021) with no indels. Such independent expansion of indels in multiple lineages and geographic locations suggests a common adaptation mechanism of SARS-CoV-2 genomes, probably to overcome host immune response, as also suggested in the recent literature (McCarthy et al., 2021; Ribes et al., 2021).

In conclusion, we conducted an in-depth analysis of indels in 4,976,200 SARS-CoV-2 genomes. We show that genomic modifications happen in a specific order, with deletions following point mutations, but growing quickly during the progress of the pandemic. In recent months we started seeing the emergence of insertions, including founder genomic modifications of the Omicron variant. Like mutations, indels are largely found in SARS-CoV-2 proteins involved in interactions with the host immune system but are preferentially located in specific regions of proteins “hypervariable regions” which overlap with structural features such as loops located close to epitopes. Indels in such regions might facilitate immune escape by remodeling the epitope surfaces and may prolong infection by these lineages. Such HVRs should be the subject of surveillance as much as common escape mutations. The increase in the number of indels and HVRs in recent lineages is likely a sign of the virus adapting to the increasing pool of resistant hosts, but other explanations, such as their role in regulating host antiviral response are also possible.

## Data Availability

All sequences used in this study are accessible via the GISAID database (www.gsaid.org). All protein structures are accessible via Protein Data Bank (https://www.rcsb.org/) and models from the Zhang lab (https://zhanglab.dcmb.med.umich.edu/COVID-19/) and AlphaFold database of COVID-19 structures (https://deepmind.com/research/open-source/computational-predictions-of-protein-structures-associated-with-COVID-19). All scripts are publicly available on GitHub repository (https://github.com/ArghavanAlisoltani/SARS-CoV-2-Indels.git).
