# Supplementary material for: Increased Frequency of Indels in Hypervariable Regions of SARS-CoV-2 Proteins—A Possible Signature of Adaptive Selection
Source: Front Genet. 2022 Jun 2;13:875406. doi: 10.3389/fgene.2022.875406 (PMC9201826; doi:10.3389/fgene.2022.875406)
Supplement: Supplementary file 1 [file Presentation1.zip › Suppl. Figures & Tables.pdf]

Supporting Information for

Increased frequency of indels in hypervariable regions of SARS-CoV-2 proteins – a possible signature of adaptive selection

Arghavan Alisoltani[1], Lukasz Jaroszewski[1], Mallika Iyer[2], Arash Iranzadeh[3], Adam Godzik[1]

[1]University of California Riverside School of Medicine, Biosciences Division, Riverside, California

[2]Graduate School of Biomedical Sciences, Sanford Burnham Prebys Medical Discovery Institute, La Jolla, CA, United States

[3]Computational Biology Division, Department of Integrative Biomedical Sciences, University of Cape Town, Cape Town, South Africa

Correspondence to: adam.godzik@medsch.ucr.edu

This file includes:

Supplementary Figures 1-4
Supplementary Tables S1 and S3

Other supplementary materials for this manuscript include the following:
Supplementary Table 2 (excel file).

A

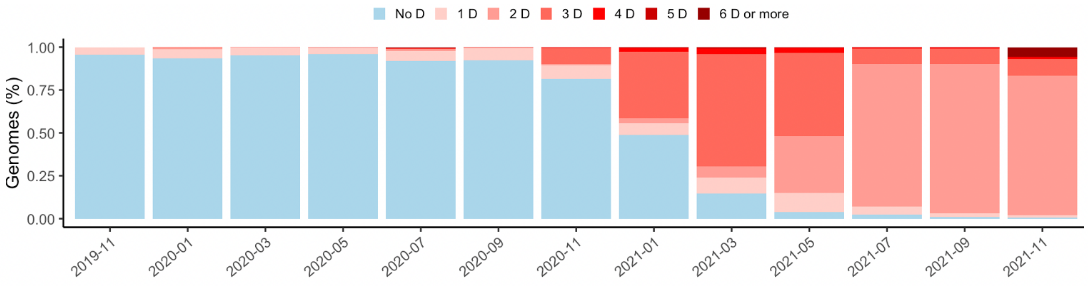

B

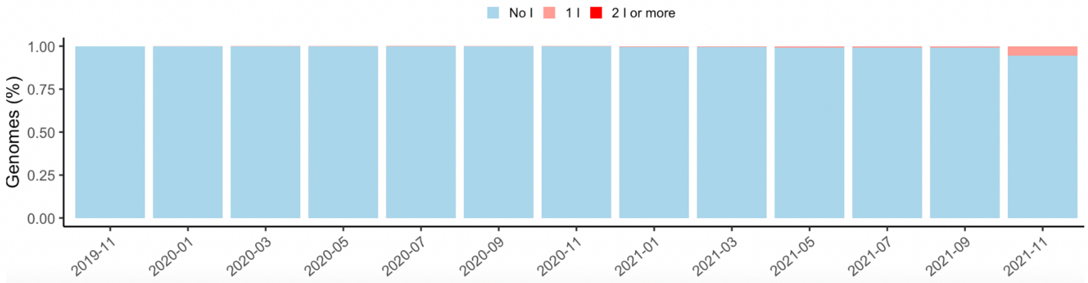

C

**Combinations of top indels**	**Number of Genomes (out of 4,9762,00 genomes)**	**Top variants**
S_156:EFR>G, 8B_119:119-120del	2531216	Delta, Gamma, Others
NSP6_106:106-108del, S_69:69-70del, S_144:144-144del	859515	Alpha, Beta, Delta, Gamma, Lambda, Others
NSP6_106:106-108del	120453	Alpha, Beta, Delta, Gamma, Lambda, Others
NSP3_1265:SL>I, NSP6_105:105-107del, S_69:69-70del, S_142:GVYY>D, S_211:NL>I, S_214:R>REPEins, N_31:31-33del	39301	Omicron, Others
8B_119:119-120del	34369	Delta, Gamma, Others
NSP1_141:141-143del, S_156:EFR>G, 8B_119:119-120del	22766	Delta, Others
NSP6_106:106-108del, S_241:241-243del	22680	Alpha, Beta, Delta, Gamma, Others
S_69:69-70del	22079	Alpha, Delta, Gamma, Others
S_156:EFR>G	19064	Delta, Gamma, Others
S_156:EFR>G, 7A_72fs, 8B_119:119-120del	15478	Delta, Others
NSP6_106:106-108del, S_69:69-70del	14650	Alpha, Delta, Gamma, Lambda, Others
NSP1_84:84-84del, S_156:EFR>G, 8B_119:119-120del	14255	Delta
NSP6_106:106-108del, S_144:144-144del	13473	Alpha, Beta, Delta, Gamma, Lambda, Others
S_69:69-70del, S_144:144-144del	11436	Alpha, Delta, Gamma, Others
S_156:EFR>G, 3A_103:A>APins, 8B_119:119-120del	10347	Delta, Others
S_156:EFR>G, 8B_86:FT>S, 8B_119:119-120del	9704	Delta, Others
NSP1_82:82-86del, S_156:EFR>G, 8B_119:119-120del	8280	Delta, Others
NSP3_1265:SL>I, NSP6_105:105-107del, S_69:69-70del, S_142:GVYY>D, S_211:NL>I, N_31:31-33del	7305	Omicron
NSP1_85:85-85del, S_156:EFR>G, 8B_119:119-120del	7243	Delta, Others
S_156:EFR>G, 7A_55fs, 8B_119:119-120del	7215	Delta, Others
NSP1_84:84-85del, S_156:EFR>G, 8B_119:119-120del	7094	Delta, Others
NSP1_82:GHVM>V, S_156:EFR>G, 8B_119:119-120del	6923	Delta, Others
S_143:V>VTins, 3A_256fs	6745	Others
NSP6_106:106-108del, S_246:RSYLTPGD>N	6240	Gamma, Lambda, Others

Supplementary Figure 1. Combinations of indels in SARS-CoV-2 variants of concern
(**A**) and **(B)** Percentage of all SARS-CoV-2 genomes with and without deletion (D) and insertion (I) events over time, respectively. **(C)** Combinations of indels in SARS-CoV-2 variants of concern obtained from GISAID as of January 7th, 2022.

A

Spike Indels	Freq	VOCs with this combination (at least 10 sequence)	HVRs
S_156:EFR>G	2791554	Delta, Gamma, Others	HVR2
S_69-70del, S_144-144del	1806862	Alpha, Delta, Gamma, Others	HVR1, HVR2
S_69-70del, S_142:GVYY>D, S_211:NL>I, S_214:R>REPEins	160392	Omicron, Others	HVR1, HVR2, HVR3
S_69-70del	40514	Alpha, Delta, Gamma, Omicron, Others	HVR1
S_144-144del	25286	Alpha, Delta, Gamma, Others	HVR2
S_241-243del	23629	Alpha, Beta, Delta, Gamma, Others	HVR4
S_69-70del, S_142:GVYY>D, S_211:NL>I	22575	Omicron	HVR1, HVR2, HVR3
S_143:V>VTins	7899	Delta, Others	HVR2
S_246:RSYLTPGD>N	6934	Lambda, Others	HVR4
S_144-144del, S_156:EFR>G	6798	Delta, Others	HVR2
S_69-70del, S_142:GVYY>D	4270	Omicron, Others	HVR1, HVR2
S_156:EF>D	4201	Delta, Others	HVR2
S_69-70del, S_156:EFR>G	4084	Delta, Omicron, Others	HVR1, HVR2
S_69-70del, S_144-144del, S_241-243del	2868	Alpha, Others	HVR1, HVR2, HVR4
S_69-70del, S_145-145del	2786	Alpha	HVR1, HVR2
S_141-143del, S_215:D>AAGYins	2474	Others	HVR2, HVR3

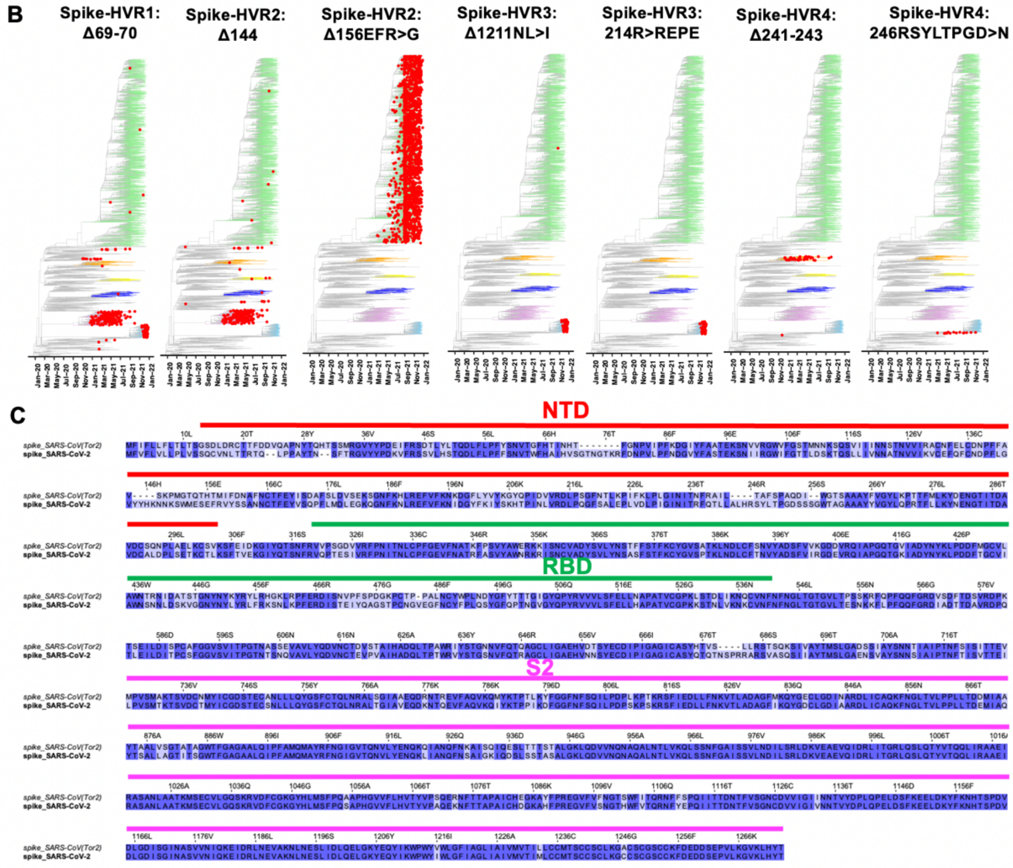

Supplementary Figure 2. **Spike glycoprotein indels.**
(**A**) Combinations of spike indels in different genomes and HVRs in spike protein of SARS-CoV-2 genomes (**B**) The most frequent indels in spike HVRs1-4 of SARS-CoV-2 shown as dots on the Nextstrain time-resolved tree, which includes 3475 genomes sampled between Dec 2019 and Dec 27th, 2021). (**C**) Spike indels compared between SARS coronavirus Tor2 (NC_004718.3) and SARS-CoV-2 (MN996527) aligned using MAFFT (default parameter) and visualized using Jalview.

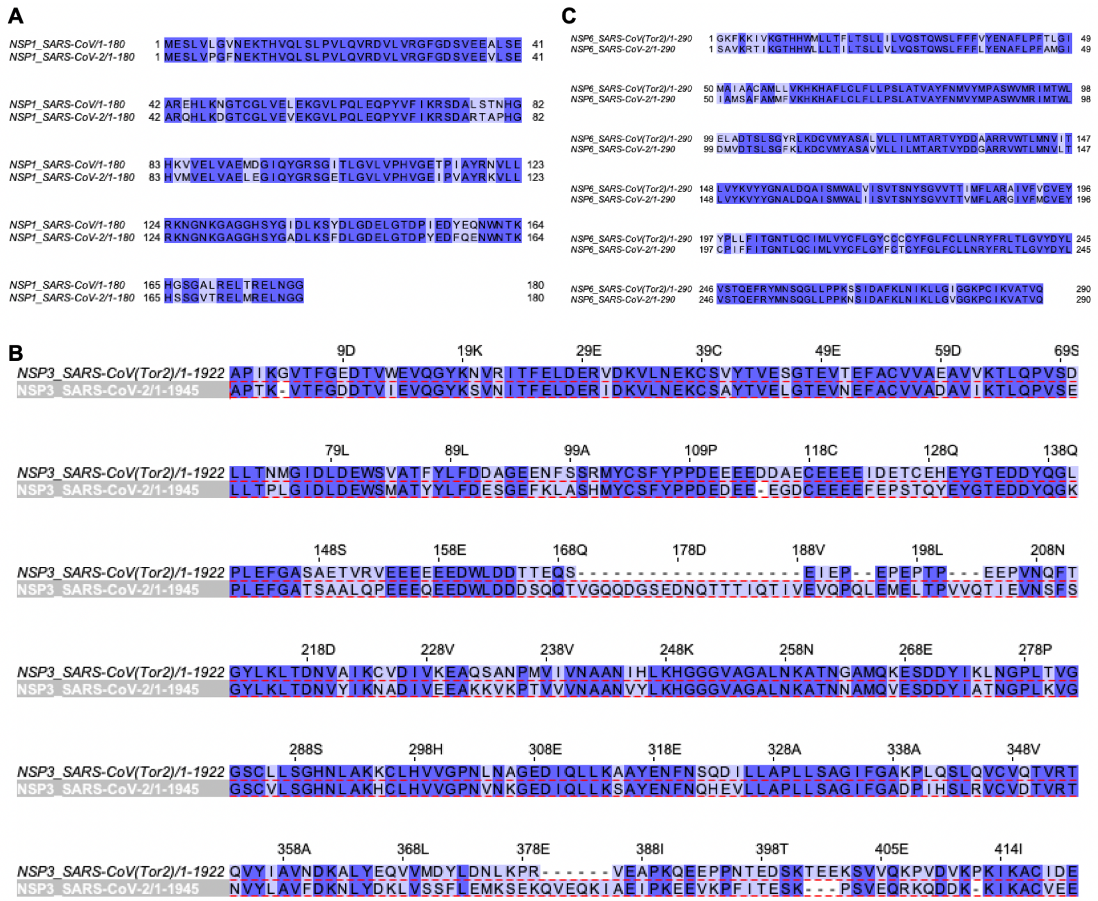

Supplementary Figure 3. Indels compared between SARS-CoV and SARS-CoV-2 proteins
Panels A to G show indels compared between SARS coronavirus Tor2 (NC_004718.3) and SARS-CoV-2 (MN996527) aligned using MAFFT (default parameter) and visualized using Jalview for each of NSP1, NSP3, NSP6, ORFs3a, 7a, 8(8b) and nucleocapsid proteins.

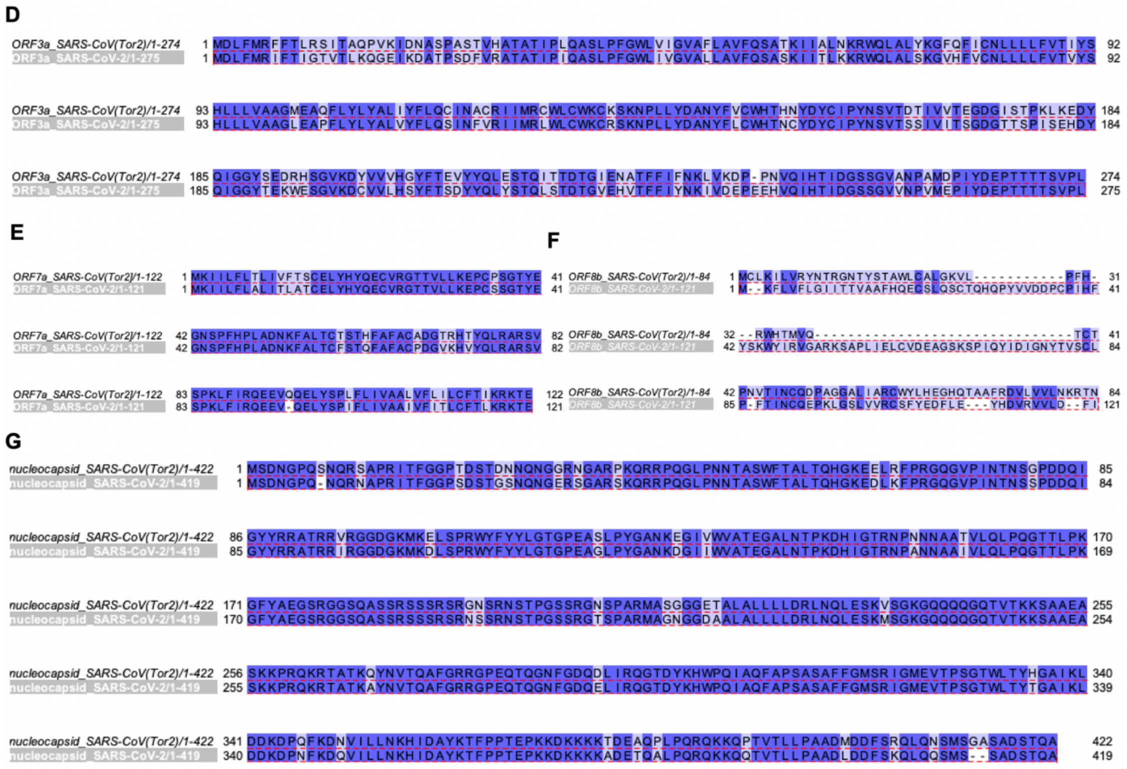

Supplementary Figure 3. Continue…

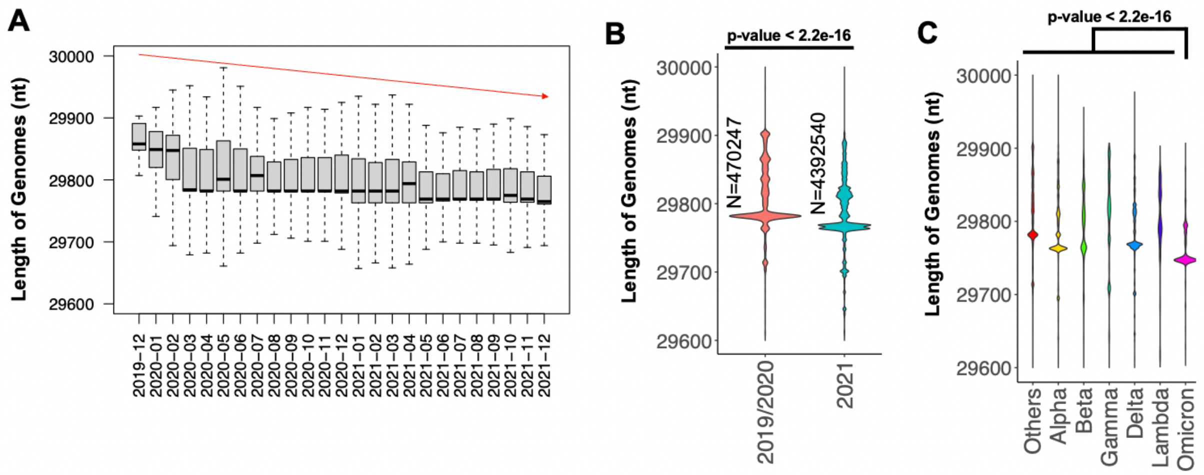

Supplementary Figure 4. SARS-CoV-2 genome shrinkage over the course of the pandemic.
(**A**) Box plots represent a shift in the length of SARS-CoV-2 genomes over the course of the pandemic (**B**) Difference in the length of SARS-CoV-2 genomes collected in 2019/2020 and 2021. (**C**) Violin plots show the variants of concern genome length. P-values calculated using Wilcoxon–Mann–Whitney test. nt: nucleotide.

Supplementary Table 1. The most frequent indels in the SARS-CoV-2 genome – the independent acquisition of indels calculated using HomoplasyFinder. Indels with minimum number of changes on tree (MNCT) above 30 and those occurred at least in two different clades (either PANGO lineages or GISAID clades) and two different time points and continents considered as potential recurrent mutations. CI: Consistency Index.

Indels	Number of genomes with indels	CI	MNCT	PANGO lineages (total 1544)	GISAID clades (total 12)	Region (total 6 continents)	Time bins (total 25 months)
S_156:EFR>G(22029:22029-22034del)	2850030	1.57E-05	63656	192	9	6	15
8B_119:119-120del(28248:28248-28253del)	2863930	2.21E-05	45328	193	11	6	15
S_144:144-144del(21991:21991-21993del)	956874	2.70E-05	37011	168	9	6	22
S_69:69-70del(21765:21765-21770del)	1024593	3.59E-05	27850	144	10	6	22
NSP6_106:106-108del(11288:11288-11296del)	1122801	3.80E-05	26304	107	8	6	21
NSP1_84:84-84del(514:514-516del)	19747	5.78E-05	17288	76	6	2	18
NSP1_141:141-143del(686:686-694del)	32524	9.79E-05	10210	210	11	6	22
NSP1_82:82-86del(508:508-522del)	13112	1.74E-04	5754	124	10	6	22
NSP1_84:84-85del(515:515-520del)	10520	1.74E-04	5732	113	10	6	22
NSP1_82:GHVM>V(510:510-518del)	10215	2.02E-04	4948	110	9	6	22
NSP1_85:85-85del(518:518-520del)	10074	2.21E-04	4526	96	9	6	22
NSP1_85:85-85del(516:516-518del)	5014	4.08E-04	2449	72	9	6	20
S_241:241-243del(22281:22281-22289del)	26795	4.33E-04	2307	37	7	6	18
7A_62:QF>H(27579:27579-27581del)	5285	5.42E-04	1845	67	7	6	18
3A_103:A>APins(25699:G>GCCC)	12327	6.34E-04	1578	46	8	6	19
8B_66:GSK>E(28090:28090-28095del)	5594	6.39E-04	1565	77	8	6	21
3A_255:255-255del(26155:26155-26157del)	4301	7.42E-04	1348	69	8	5	21
S_156:EF>D(22030:22030-22032del)	4202	7.78E-04	1285	11	2	2	4
NSP1_85:MVE>K(519:519-524del)	1897	8.24E-04	1214	41	8	6	17
S_156:EF>V(22029:22029-22031del)	1263	8.83E-04	1132	29	2	3	7
S_243:243-244del(22289:22289-22294del)	2184	1.01E-03	994	44	7	6	18
NSP1_86:86-86del(521:521-523del)	1465	1.09E-03	915	43	8	5	16
N_214:214-215del(28912:28912-28917del)	3236	1.15E-03	871	43	4	5	16
S_143:V>VTins(21990:T>TTAC)	8146	1.16E-03	864	6	5	3	9
NSP1_84:84-86del(514:514-522del)	1562	1.20E-03	836	40	8	5	15
8B_119:119-119del(28248:28248-28250del)	845	1.23E-03	814	11	2	3	7
NSP1_81:81-82del(505:505-510del)	2611	1.31E-03	764	29	6	5	16
8B_118:118-119del(28244:28244-28249del)	1159	1.54E-03	650	24	4	3	8
7B_13:13-13del(27792:27792-27794del)	2454	1.54E-03	649	41	7	6	17
7A_101:101-102del(27692:27692-27697del)	2526	1.76E-03	569	22	5	6	12
S_143:VY>D(21990:21990-21992del)	797	1.79E-03	560	10	5	4	14
8B_118:LDFI>F(28246:28246-28254del)	792	1.84E-03	544	24	2	5	9
NSP1_54:54-54del(423:423-425del)	756	1.93E-03	517	23	5	4	15
S_141:141-143del(21981:21981-21989del)	2140	2.00E-03	499	13	6	6	14
NSP3_1263:1263-1263del(6506:6506-6508del)	1374	2.02E-03	495	23	6	5	17
3A_256:VN>D(26159:26159-26161del)	1404	2.07E-03	484	34	7	4	18
S_210:210-210del(22189:22189-22191del)	2685	2.12E-03	472	34	7	6	18
3A_103:A>APPins(25699:G>GCCCCCC)	1748	2.17E-03	461	6	4	3	6
NSP2_268:268-268del(1605:1605-1607del)	1752	2.18E-03	459	26	7	6	20
S_141:141-144del(21982:21982-21993del)	946	2.25E-03	444	24	7	5	22
S_156:156-157del(22028:22028-22033del)	540	2.35E-03	425	19	2	4	11
NSP3_276:NG>R(3546:3546-3548del)	511	2.51E-03	398	18	6	4	16
NSP3_1265:SL>I(6513:6513-6515del)	52360	2.69E-03	372	27	8	6	13
S_145:145-145del(21995:21995-21997del)	1627	2.70E-03	370	3	3	3	8
NSP6_105:105-107del(11283:11283-11291del)	51523	2.72E-03	368	15	7	6	13
7A_103:103-103del(27698:27698-27700del)	869	2.78E-03	360	16	6	5	14
N_2:SD>Y(28278:28278-28280del)	5538	2.82E-03	355	3	4	5	9
6_2:2-2del(27205:27205-27207del)	5568	2.93E-03	341	4	4	5	11
7A_102:102-103del(27695:27695-27700del)	992	2.95E-03	339	17	7	4	15

S_143:143-143del(21988:21988-21990del)	1138	3.03E-03	330	5	5	4	11
S_215:D>AAGYins(22205:G>GCGGCAGGCT)	1526	3.15E-03	317	4	2	3	10
7B_14:14-14del(27795:27795-27797del)	1879	3.38E-03	296	23	7	5	14
NSP3_1323:1323-1323del(6684:6684-6686del)	664	3.48E-03	287	18	4	3	11
7A_110:110-110del(27721:27721-27723del)	736	3.51E-03	285	19	5	4	13
8B_86:FT>S(28150:28150-28152del)	10635	3.57E-03	280	5	3	4	8
7A_59:59-62del(27568:27568-27579del)	2284	3.69E-03	271	15	7	4	13
NSP14_287:287-287del(18896:18896-18898del)	594	3.69E-03	271	18	6	4	13
7A_104:104-104del(27701:27701-27703del)	730	3.79E-03	264	20	6	4	10
8B_58:58-58del(28065:28065-28067del)	692	3.86E-03	259	19	5	4	14
S_143:VYY>D(21990:21990-21995del)	715	3.91E-03	256	21	6	4	15
S_211:NL>I(22194:22194-22196del)	49514	3.91E-03	256	20	8	6	13
NSP2_265:265-266del(1598:1598-1603del)	833	4.12E-03	243	16	6	4	19
7A_75:YQL>*(27618:27618-27623del)	624	4.31E-03	232	18	4	4	11
3A_257:257-259del(26161:26161-26169del)	563	4.42E-03	226	15	6	4	12
7A_100:100-102del(27691:27691-27699del)	666	4.48E-03	223	16	5	4	11
NSP1_135:SY>N(669:669-671del)	576	4.61E-03	217	15	5	4	15
NSP1_82:82-85del(509:509-520del)	564	5.26E-03	190	15	4	4	8
N_208:AR>G(28896:28896-28898del)	5615	5.41E-03	185	12	5	6	13
S_142:GVYY>D(21987:21987-21995del)	50996	5.88E-03	170	13	6	6	9
S_246:RSYLTPGD>N(22299:22299-22319del)	7070	6.37E-03	157	2	3	4	11
NSP3_411:DK>E(3951:3951-3953del)	581	6.45E-03	155	13	6	4	14
3A_256:256-259del(26158:26158-26169del)	596	6.54E-03	153	17	6	5	16
NSP6_104:SL>M(11283:11283-11285del)	1539	7.25E-03	138	14	6	3	13
3A_19:19-28del(25446:25446-25475del)	1065	8.20E-03	122	4	3	4	8
8B_66:66-67del(28087:28087-28092del)	793	8.47E-03	118	15	5	4	11
NSP3_1201:1201-1201del(6320:6320-6322del)	638	9.01E-03	111	5	3	3	6
7B_44:*TNMKF>Y(27887:27887-27901del)	5337	9.35E-03	107	9	3	6	11
8B_45:WY>C(28028:28028-28030del)	1139	9.35E-03	107	8	3	3	11
8B_66:GS>A(28090:28090-28092del)	500	1.01E-02	99	12	4	4	15
6_22:22-30del(27264:27264-27290del)	894	1.06E-02	94	32	5	4	17
S_214:R>RTDRins(22204:T>TACAGATCGA)	1051	1.11E-02	90	3	2	3	6
3A_29:29-29del(25475:25475-25477del)	1273	1.14E-02	88	13	3	4	9
7A_54:54-62del(27551:27551-27577del)	2507	1.35E-02	74	17	4	3	9
NSP3_206:206-207del(3335:3335-3340del)	981	1.37E-02	73	7	4	2	10
N_31:31-33del(28362:28362-28370del)	50567	1.47E-02	68	6	3	6	3
S_214:R>REPEins(22204:T>TGAGCCAGAA)	41221	1.47E-02	68	4	2	6	3
NSP3_1313:1313-1321del(6655:6655-6681del)	719	1.85E-02	54	19	5	3	13
10_9:9-10del(29582:29582-29587del)	1276	2.27E-02	44	2	3	2	7
7A_59:59-64del(27568:27568-27585del)	557	2.70E-02	37	18	6	5	11
7A_54:FALTCFSTQ>L(27555:27555-27578del)	1806	2.78E-02	36	36	6	4	12

Supplementary Table 2.

Supplementary Table 2 is a separate excel spreadsheet file containing sub-tables S2a to S2i. This table include information on independent co-occurrences and correlations of indels in variants of concerns.

Supplementary Table 3. Structures, models, and other details of protein functional regions used in Figures 2 and 3 in this paper. Structural models for visualization of HVRs on NSP3, NSP6, ORF3a, and nucleocapsid protein 3D structures obtained from https://zhanglab.ccmb.med.umich.edu/COVID-19/. PDB: Protein Data Bank; DB: database

Protein	Annotation	Start	End	Annotation/Source	PDB IDs
ORF3a	inside	57	76	UniProt DB/ PDB/TMHMM server	6xdcA
ORF3a	inside	126	275	UniProt DB/ PDB/TMHMM server	6xdcA
ORF3a	outside	1	33	UniProt DB/ PDB/TMHMM server	6xdcA
ORF3a	outside	100	102	UniProt DB/ PDB/TMHMM server	6xdcA
ORF3a	TMhelix	34	56	UniProt DB/ PDB/TMHMM server	6xdcA
ORF3a	TMhelix	77	99	UniProt DB/ PDB/TMHMM server	6xdcA
ORF3a	TMhelix	103	125	UniProt DB/ PDB/TMHMM server	6xdcA
ORF7a	SP	1	15	UniProt DB/ PDB/TMHMM server	6xdcA
ORF7a	Ig-like ectodomain	16	96	UniProt DB/ PDB/(1)	6w37A
ORF7a	TMhelix	97	116	UniProt DB/ PDB/(1)	6w37A
ORF7a	typical ER retention motif	117	121	UniProt DB/ PDB/(1)	6w37A
ORF8	SP	1	15	UniProt DB/	7jx6A
nsp1	nsp1 head, N-terminal domain	1	112	UniProt DB/	7k3nA
nsp1	nsp1 linker	113	151	UniProt DB	NA
nsp1	plug domain, C-terminal domain	152	179	UniProt DB/ PDB	7k5i1
nsp2		1	638	PDB	7MSW
nsp3	UBL1	1	107	UniProt DB/ PDB	7kagA
nsp3	E/Q-rich region	111	206	UniProt DB	NA
nsp3	ADRP	208	372	UniProt DB/ PDB	6w02A
nsp3	SUD	413	676	UniProt DB/ PDB	2w2gA
nsp3	SUD-C	679	743	UniProt DB/ PDB	2kafA
nsp3	PlPro	748	1060	UniProt DB/ PDB	6w9cA
nsp3	interdomain linker	1061	1088	UniProt DB	NA
nsp3	NAB	1089	1203	UniProt DB/ PDB	2k87A
nsp3	G2M	1204	1412	UniProt DB	NA
nsp3	Ectodomain	1436	1500	UniProt DB	NA
nsp3	TMhelix	1413	1435	UniProt DB	NA
nsp3	TMhelix	1501	1584	UniProt DB	NA
nsp6	inside	1	11	TMHMM server	NA
nsp6	inside	61	66	TMHMM server	NA
nsp6	inside	133	138	TMHMM server	NA
nsp6	inside	180	183	TMHMM server	NA
nsp6	inside	233	290	TMHMM server	NA
nsp6	outside	32	40	TMHMM server	NA
nsp6	outside	90	112	TMHMM server	NA
nsp6	outside	159	161	TMHMM server	NA
nsp6	outside	207	209	TMHMM server	NA
nsp6	TMhelix	12	31	TMHMM server	NA
nsp6	TMhelix	41	60	TMHMM server	NA
nsp6	TMhelix	67	89	TMHMM server	NA
nsp6	TMhelix	113	132	TMHMM server	NA
nsp6	TMhelix	139	158	TMHMM server	NA
nsp6	TMhelix	162	179	TMHMM server	NA
nsp6	TMhelix	184	206	TMHMM server	NA
nsp6	TMhelix	210	232	TMHMM server	NA
S	SP	1	12	UniProt DB/ PDB	7KNB
S	NTD	13	303	UniProt DB/ PDB	71qwA/7KNB
S	RBD	319	541	UniProt DB/ PDB	6lzgB/7KNB
S	S2 subunit	710	1274	UniProt DB/ PDB	7KNB
S	TMhelix	1214	1236	UniProt DB/ PDB	7KNB

S	**RBM**	**437**	**508**	**UniProt DB/ PDB**	**7KNB**
N	**NTD**	**48**	**174**	**UniProt DB/ PDB**	**6m3mA/7KNB**
N	**CTD**	**247**	**364**	**UniProt DB/ PDB**	**6wjiA/7KNB**
N	**RBD**	**41**	**186**	**UniProt DB/ PDB**	**7KNB**
N	**Dimerization**	**258**	**361**	**UniProt DB/ PDB**	**7KNB**

1. Z. Zhou *et al.*, Structural insight reveals SARS-CoV-2 ORF7a as an immunomodulating factor for human CD14(+) monocytes. *iScience* **24**, 102187 (2021).
